# Supplementary material for: Prioritizing Disease Diagnosis in Neonatal Cohorts through Multivariate Survival Analysis: A Nonparametric Bayesian Approach
Source: Healthcare (Basel). 2024 May 2;12(9):939. doi: 10.3390/healthcare12090939 (PMC11083100; doi:10.3390/healthcare12090939)
Supplement: Supplementary file 1 [file healthcare-12-00939-s001.zip › healthcare-2996452-supplementary.pdf]

Supplementary Materials

Prioritizing Disease Diagnosis in Neonatal Cohorts Through Multivariate Survival Analysis: A Nonparametric Bayesian Approach

Jangwon Seo[1], Junhee Seok[1], Yoojoong Kim[2,*]

[1]School of Electrical Engineering, Korea University, Seoul 02841, Republic of Korea; jwein307@korea.ac.kr (J.S.); jseok14@korea.ac.kr (J.S.)

[2] School of Computer Science and Information Engineering, The Catholic University of Korea, Bucheon 14662, Republic of Korea

[*] Corresponding authors

Email addresses:

yoojoongkim@catholic.ac.kr,

Table

Table S1. This table illustrates the categorization of diseases into groups based on similarities, as defined by the major classifications of the ICD-10. Supplementary Table S1 presents the disease codes, incidence rates, and descriptions for the 27 disease categories classified according to the ICD-10 major classification.

ICD (27)	Diagnosis rate (%)	Description
A00-A99	82.6%	Certain infectious and parasitic diseases
B00-B99	78.35%	
C00-D48	0.26	Malignant neoplasms
D50-D-89	9.21	Diseases of the blood and blood-forming organs and certain disorders involving the immune mechanism
E00-E90	5.61	Endocrine, nutritional and metabolic diseases
F00-F99	8.22	Mental and behavioural disorders
G00-G99	7.12	Diseases of the nervous system
H00-H59	86.39	Diseases of the eye and adnexa
H60-H95	81.47	Diseases of the ear and mastoid process
I00-I99	3.58	Diseases of the circulatory system
J00-J99	99.87	Diseases of the respiratory system
K00-K93	82.07	Diseases of the digestive system
L00-L99	92.76	Diseases of the skin and subcutaneous tissue
M00-M99	23.57	Diseases of the musculoskeletal system and connective tissue
N00-N99	29.48	Diseases of the genitourinary system
O00-O99	0.06	Pregnancy, childbirth and the puerperium
P00-P96	9.79	Certain conditions originating in the perinatal period
Q00-Q99	8.34	Congenital malformations, deformations and chromosomal abnormalities
R00-R99	61.80	Symptoms, signs and abnormal clinical and laboratory findings, not elsewhere classified
S00-S99	66.21	Injury, poisoning and certain other consequences of external causes
T00-T98	34.36	
U00-U85	0	Codes for special purposes
V01-V99	0	External causes of morbidity and mortality

W00-W99	0.37	
X00-X99	0.07	
Y00-Y98	0.06	
Z00-Z99	8.39	Factors influencing health status and contact with health services

Table S2. Top six frequent sequences of events based on the precedence and sequence analysis score function for the observed 4343 patients. The most probable sequence order with the least score was respiratory, skin, infectious, digestive, ear, eye, and, injury diseases. Sequences with scores not more than 2% higher than the best score were above the borderline as valid sequences.

**Event Sequence**	**Score**	**Rise Rate**
Respiratory→Skin→Infectious→Digestive→Ear→Eye→Injury	6.85	0.00
Respiratory→Infectious→Skin→Digestive→Ear→Eye→Injury	6.89	0.51
Respiratory→Skin→Infectious→Ear→Digestive→Eye→Injury	6.89	0.51
Respiratory→Infectious→Skin→Ear→Digestive→Eye→Injury	6.92	1.01
Respiratory→Skin→Infectious→Digestive→Eye→Ear→Injury	6.98	1.79
Respiratory→Skin→Infectious→Digestive→Ear→Injury→Eye	7.01	2.29

Figures

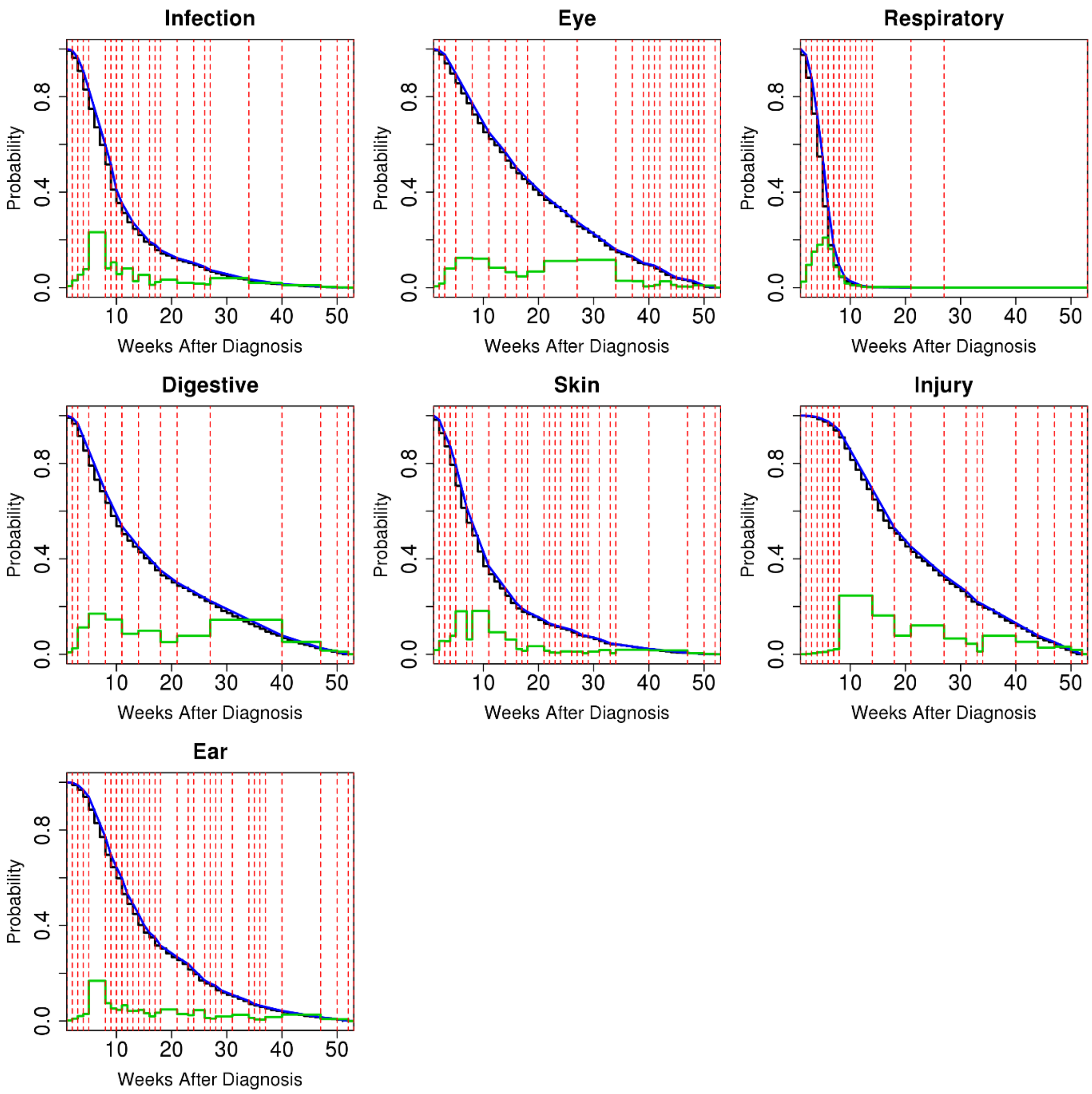

Figure S1. Univariate analyses of single events for the observed 4343 patients. The probability of the event occurrence was estimated for each disease diagnosis event, by CEPA (blue) and the well-known Kaplan–Meier (black) method. The dashed red vertical lines represent the CEPA partitions within which the probability density was expected to be uniform. The green lines represent the probability masses assigned to the CEPA partitions.

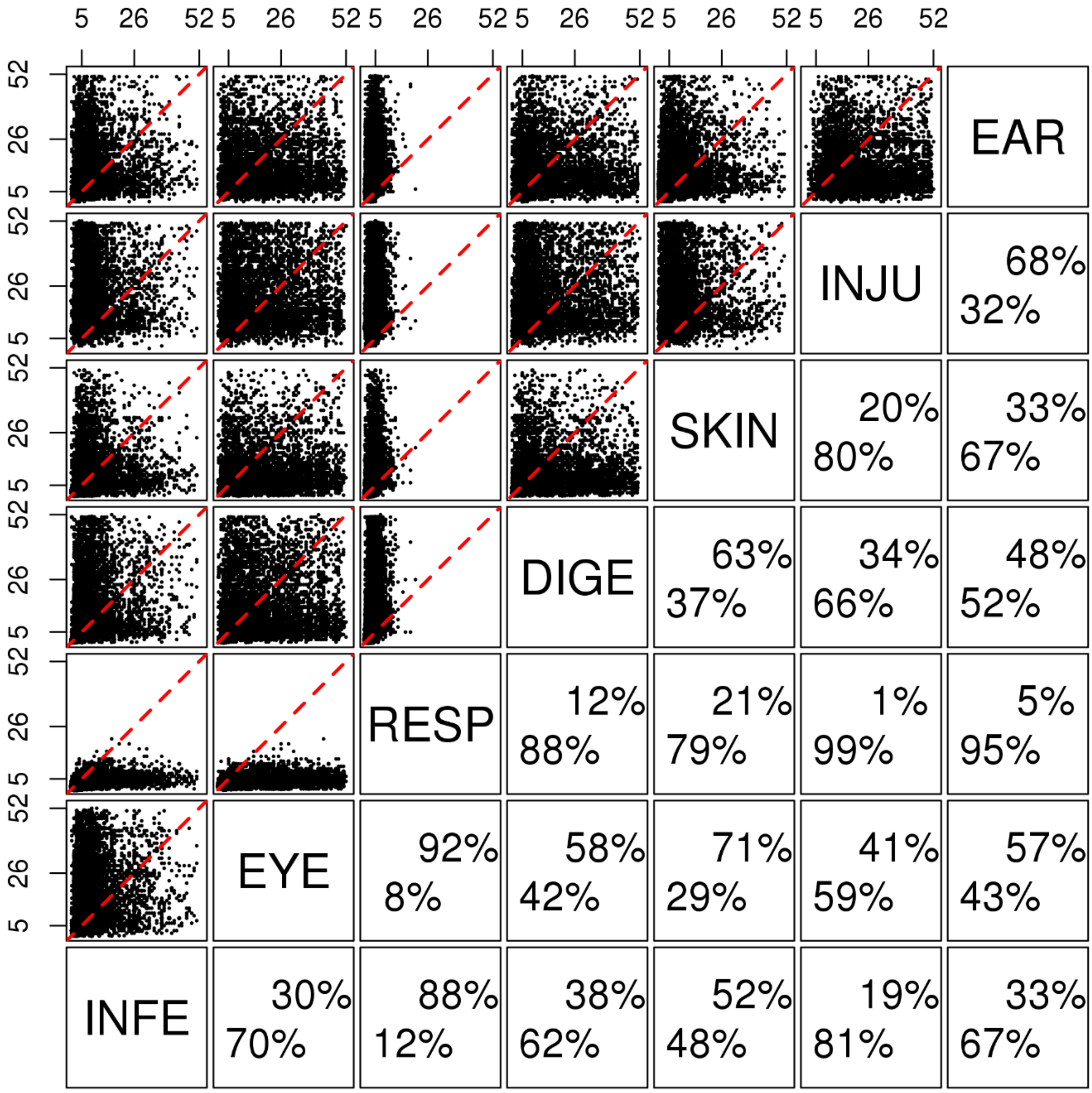

Figure S2. Pairwise precedence of the disease diagnosis events for the observed 4343 patients. A joint distribution of bivariate time-to-events was estimated by CEPA for each pair of events. The considered events were the diagnoses of infectious disease (INFE), eye disease (EYE), respiratory disease (RESP), digestive disease (DIGE), skin disease (SKIN), injury (INJU), and ear disease (EAR). Each top-left panel shows the observed or censored days of bottom and right-side events. Each bottom-right panel shows the precedence chance of the top-side to the left-side events (top-right %) and that of the opposite case (bottom-left %) when both events occurred at different times. For example, respiratory diseases preceded digestive diseases and were diagnosed 88% of the time.
